# Supplementary material for: Are carbon emissions trading and green financial instruments synergistic? -Comprehensive quantitative research based on content analysis
Source: PLoS One. 2024 Mar 7;19(3):e0298601. doi: 10.1371/journal.pone.0298601 (PMC10919601; doi:10.1371/journal.pone.0298601)
Supplement: S1 Data — (ZIP) [file pone.0298601.s001.zip › Data/地级市Z_F工作报告.zip!/╣π╢1⁄2/╘╞╕í╩╨/└┤╫╘╣1⁄2╓┌║┼í╛┬φ┐╦╩2╛▌═°í┐_2013.docx]

各位代表：

我代表市人民政府向大会作政府工作报告，请予审议，并请政协各位委员和其他列席人员提出意见。

2012年工作回顾

过去一年，是我市发展承前启后、继往开来的重要一年,也是深入贯彻落实科学发展观、全面实施“十二五”规划的关键之年。市政府在省委、省政府和市委的正确领导下，在市人大及其常委会和市政协的监督支持下，围绕“跨越赶超、科学崛起”和建设幸福云浮目标，带领全市人民，抢抓机遇、团结实干，顺利完成了市五届人大一次会议确定的各项目标任务。

一、发展速度进一步加快

大力实施“集聚带动、转型拉动、创新驱动、统筹推动”发展战略，在全球经济呈现趋势性下滑，国内经济增长放缓的情况下，我市经济保持稳步增长的良好态势，发展速度进一步加快。2012年全市实现地区生产总值540.45亿元，比上年（下同）增长12.8%；人均生产总值增长11.5%；规模以上工业增加值138.76亿元，增长26.1%；地方公共财政预算收入36.76亿元，增长23.4%；固定资产投资总额463.66亿元，增长31.3%；社会消费品零售总额180.31亿元，增长11.6% ;外贸进出口总额14.57亿美元，增长4.8%；实际吸收外商直接投资1.06亿美元，增长15.4%；12月末，金融机构本外币存款余额658.93亿元、贷款余额395.46亿元，分别增长13.3%和18.7%。主要经济指标增速在全省排位靠前，地区生产总值和固定资产投资增速均排全省第3位，规模以上工业增加值和地方公共财政预算收入增速均排全省第2位。

二、发展方式进一步转变

产业结构不断优化。五大百亿特色产业逐步走上规范化、集群化发展轨道，产业链不断延伸，五大产业增加值占规模以上工业增加值比重为54.3%。第九届中国（云浮）国际石材科技展览会暨第三届中国（云浮）石文化节圆满成功，参展企业和专业观众、贸易成交额和展会影响力均创新高。投资50亿元的中国•云浮国际石材产业城加快建设，石材产业集聚发展、结构调整及升级改造又一基础平台即将建成。清洁能源开发和利用迈出了新步伐，物联网、文化创意、健康养生等新兴产业正在兴起，新业态逐渐形成。全市一、二、三产业所占GDP比重由2011年的24.9：42.5：32.6调整为2012年的23.9：44.2：31.9。

重点项目建设发力提速。全市100项重点工程项目累计完成投资188.39亿元，占年计划投资的98.2%。列入省的19项重点建设项目累计完成投资59.95亿元，完成年度计划的108.9%。引进了碧桂园养生谷及房地产、康利（云浮）石材工业产业园以及珠海华策半导体LED产业基地等一批投资均超30亿元的大项目。2012云浮•大西关建设经贸活动全市签约、动工、竣工项目232个，总投资1200.53亿元。恒星高新科技、高丘六和、广东广云铝材等一批重大项目建成投产，中材罗定水泥、华润罗定水泥、云浮亚婆髻风电、新兴天露山风电等一批重大项目加快推进。

“双转移”工作成效明显。全市招商引资合同项目291个，合同投资总额770.5亿元，其中投资10亿元以上项目22个。累计承接产业转移项目495个，总投资575.55亿元。工业园区建设加快，佛山（云浮）产业转移工业园确立了“园镇统合发展”模式，云浮（罗定）循环经济产业基地（双东环保工业园）被认定为省市共建基地，云浮循环经济工业园正在申报部省共建工业园。“四大工业园区”基础设施建设累计投入68.49亿元，引入项目395个。两个省级产业转移工业园实现规模以上工业增加值20.38亿元。统筹推进城乡就业工作，新增城镇就业3.85万人，新增农村劳动力转移6.11万人，组织农村劳动力技能培训3.8万人，培训后转移就业率89.8%。开展“创业之星”评选活动，实施“千名企业家创业培训公益工程”和“百名残疾人创业就业帮扶工程”，促进创业带动就业2865人，新兴县被评为全省“创业先进城市”。

农村经济持续稳步增长。实现农林牧渔业总产值206.67亿元，增长6%。农民人均纯收入9219元，增长13.2%。农业产业化组织673个，销售总收入达396.5亿元，增长8.1%，带动农户27.3万户，户均参与产业化经营收入9150元，增长7.6%。新登记农民专业合作社255户，增长106%。国家级农业龙头企业温氏集团全年销售收入达335亿元。无公害农产品、绿色食品和有机食品生产基地发展到57个，认证产品101个。粮食生产继续获得丰收，完成省下达考评任务。

现代服务业蓬勃发展。现代物流、商贸流通、金融保险、休闲旅游、住宿餐饮、文化娱乐等服务业加快发展，第三产业增加值172.53亿元，占GDP比重31.9%。商务流通环境进一步优化，家乐福、东方时代广场等重点项目建设不断加快。新建平价商店77家，完成省下达任务的154%。金水台太阳岛水上乐园、罗定国际大酒店等一批旅游接待新项目建成投入使用。云浮国际石材博览中心、新兴天露山旅游度假区分别成功创建省工业旅游和省旅游示范基地。全市旅游接待游客1123.8万人次、旅游总收入124.94亿元，同比增长40.2%和44.5%。

科技创新和品牌建设扎实推进。获批立项省级以上科技项目累计80项，其中产学研合作项目27项。创建省级以上工程技术研究开发中心1个，组建各类创新平台6个、研发机构1所。全市获省级名牌产品共16个、强制性产品认证企业18家、管理体系认证企业104家、食品农产品认证企业39家。“云浮市石材商会”和“新兴县不锈钢商会”两个集体商标获国家批准注册，实现集体商标零突破。“托洞腐竹”、“新兴排米粉”成为新的地标保护产品，全市获地标保护产品达到11个，位居全省地级市前列。

三、发展环境进一步优化

城乡交通明显改善。全力推进以“七三三三一”工程为重点的交通基础设施建设，全年投入交通基础设施建设资金50.08亿元。云罗高速双凤至双东段、沿江公路建成通车，省道113线、省道276线等路面大修项目完成主体工程。江罗高速动工建设，南广高铁云浮段、阳罗高速加快建设，汕湛高速清远至新兴段完成工可报告修编。云浮港总体规划通过省政府审批。罗信高速、怀罗高速、广梧高速思劳立交东移和高村立交、南江口码头等项目前期工作加快推进。建设农村公路416公里，实现全市100%乡镇有客运站、符合条件的行政村100%通客车和100%有候车亭的目标。

人居环境建设有新突破。实施市城区扩容提质工程，启动云浮新区规划工作，圆满完成西江新城、中央商务区、行政中心规划国际竞赛。启动了英东体育馆广场地下人防商城项目的前期工作，蟠龙天湖片区升级改造项目动工建设。加快“三旧”改造和恒大城、碧桂园、臻汇园等房地产开发建设，房地产开发投资31.97亿元，同比增长35.99%。扎实推进竞争性“以奖代补”和村级公益事业一事一议财政奖补项目，建成项目1970个，惠及群众115万人。建成名镇名村18条、示范村28条，宜居城镇6个、宜居村庄86条、宜居社区3个，省级卫生村30条。其中，罗定市罗镜镇成为第一批“广东省宜居示范城镇”，云城区下白村、新兴县龙山塘村等8个村成为第一批“广东省宜居示范村庄”。创建国家卫生城市工作深入推进，成功创建广东省园林城市。我市被定为全省山区唯一的“智慧城市”试点市。

生态文明建设亮点纷呈。国土资源管理水平不断提高，顺利通过了省政府对我市履行耕地保护目标责任制考核，连续12年实现耕地占补平衡。争取国家、省财政林业建设资金共1.496亿元，完成造林作业面积11.96万亩、林业生态文明“万村绿”村庄建设107个、森林抚育作业面积30.79万亩，全市森林覆盖率67.8%。全面完成了林权确权工作。全市城镇生活污水集中处理率68%，市城区生活垃圾无害化处理率100%，城区降尘量年均值同比下降6.1%，农村污水处理率39%，饮用水源水质和西江云浮段水质均保持二类标准，集中式饮用水源水质达标率和省控断面水质达标率100%。全年空气质量优良率达100%，全优天数比例达80%以上。区域环境噪声等效声级年均值均达一类区标准。全市91个减排项目完成率为100%，污染减排工作的经验做法得到省的充分肯定。

改革创新深入推进。农村综合改革有序推进，积极开展现代农业“公司+理事会+农户、合作社+合作社、公司+现代家庭农场”三种机制的探索。以农村金融改革为切入点，大胆探索创建征信中心和信用村、实施金融扶贫、开展农村建房贷款试点、推广银行卡助农取款点和征信数据应用等，建立“政府引导、市场运作”征信体系，农村商业银行、村镇银行、小额贷款公司、融资担保公司等新型农村金融机构（组织）有新发展。我市被确定为全省“农村金融改革创新综合试验区”和“社会信用体系建设试点市”，获得省政府颁发“2012年金融稳定奖”。信用云浮建设、培育和发展自然村乡贤理事会、弘扬优秀传统文化促进社会管理创新、石材企业法律服务中心等4个项目被省确定为社会创新观察项目。建立健全了县（市、区）、镇（街）、村（居）、村民小组的四级调解网络，并发挥社会组织的积极作用，由人民调解、司法调解、行政调解向中介、行业、群团调解拓展，构建了上下联调联动的新模式。

四、社会保障体系进一步健全

民生保障水平不断提高。全市11大类民生支出完成65.48亿元，增长27.49%，占公共财政预算支出比重的70.9%。其中，投入24.75亿元推进市十件民生实事建设。圆满完成扶贫开发“双到”工作任务，87个贫困村，有劳动能力的10161户44111人全部脱贫，3300户农村低收入住房困难户住房改造建设任务全面完成。10.4万人饮水不安全问题列入省农村饮水安全工程新增规划。投入水利建设资金6.36亿元，推进44宗水利项目建设。罗定市被评为全国农田水利基本建设先进单位。医药卫生体制改革继续深化，公共卫生和基本医疗服务能力明显提升。全面完成30间基层医疗卫生机构升级改造和1.5万户农村改厕工作。市人民医院新院首期工程和云安县人民医院新院建成投入使用。我市被评为全国基层（社区和农村）中医药工作先进单位。罗定市人民医院顺利通过省的三级甲等医院评审验收，实现我市三级甲等医院零的突破。城乡居民社会养老保险和基本医疗保险实现了一体化、全覆盖，被省确定为城乡居民基本医疗保险引入市场机制试点市，对参保人实行“二次补偿”和“大病保险”，大大减轻参保人医疗负担，试点成效得到了国家和省主管部门的充分肯定。新兴县人力资源和社会保障局被评为全国新型农村和城镇居民社会养老保险工作先进单位。加载金融功能的社会保障卡成功发行，医疗保险诊疗实现市内异地实时结算。提高了低保标准，出台了80岁以上困难老人发放津贴政策，落实了孤儿基本生活保障制度。开工建设保障性住房1141套，竣工722套。在全市开展农房统保，成为全省首个行政性农房统保区。全市继续保持人口低生育水平，完成人口和计划生育管理目标任务。

教育文化强市工作取得新成效。15个镇（街）教育创强工作顺利通过省督前检查，已有8个镇（街）通过督导验收。全市义务教育规范化学校覆盖率达57.57%。高中布局调整和一批校舍安全改造项目加快建设，被评为“高中阶段教育普及市”。市中专学校新校区投入使用，华立城市学院、市技工学校新校区建设加快推进，职业教育基础能力不断增强。文化体制改革任务全面完成，提前完成市本级政府机关软件正版化工作。第三届石文化节顺利升格为中国（云浮）石文化节。“云浮石艺”和“禾楼舞”传承基地被定为省级非遗传承基地。建成镇（街）农民体育健身广场12个，村文体广场100个。全市公共文化服务水平在全省落实科学发展观量化指标考核中排名第七位。

平安云浮建设深入推进。抓好“三打两建”工作，全面打击欺行霸市、制假售假、商业贿赂领域的违法犯罪行为，深入开展打私、打传、产品质量和食品药品安全专项整治。全市共查处案件14061宗，涉案金额（货值）4.89亿元，市场秩序不断规范。开展打击“三违三抢”行为活动，查处案件3378宗。认真开展基层大接访活动，一批信访积案和遗留问题得到解决，信访维稳工作得到加强，社会矛盾及时有效化解。强化安全生产专项整治，完成省下达的控制指标任务，安全生产形势总体平稳。进一步完善应急管理机制，预防和应对突发事件能力有效提升。劳动者合法权益得到切实保障，和谐劳动关系得到新发展。

五、政府效能建设进一步加强

坚持依法行政，认真执行市人大及其常委会各项决议并报告工作，主动向市政协通报情况，广泛听取民主党派、工商联、无党派人士和各人民团体意见，共办理人大代表建议89件、政协委员提案161件。切实转变政府职能，全面开展富县强镇、简政强镇事权改革，县级和镇级大部制改革全面实施。市直事业单位分类改革圆满完成，县（市、区）的改革也基本完成。大力推进行政审批制度改革，压减行政审批事项366项，占全部事项的48.03%。完善政府决策机制，出台并实施了《云浮市重大行政决策专家咨询办法实施细则（试行）》。各级行政服务中心和行政电子监察系统进一步完善，市网上办事大厅开通运行。挂钩联系、倒逼和督查通报机制进一步健全，工作作风实现新转变，干部执行力进一步增强。效能监察、廉政建设和反腐败工作进一步加强，查处了一批违纪违法案件。

此外，物价、统计、人事人才、经济发展研究、知识产权、项目代建服务、民族宗教、档案、外事侨务、港澳事务、台务、人民防空、地方志、消防、保密、气象、侨联、文联、社科联、妇儿、工商联、红十字会、贸促会、新闻出版以及驻市外机构等工作也取得新成绩。

各位代表！过去一年政府工作取得的成绩，来之不易。这是在省委、省政府和市委的正确领导下，全市上下开拓进取、奋力拼搏的结果。在此，我代表市人民政府，向全市人民、驻云浮部队官兵和关心支持云浮发展的港澳台同胞、海外侨胞及国际友人表示衷心的感谢并致以崇高的敬意！

同时，我们也清醒地看到，我市经济社会发展还存在一些亟待解决的突出问题。主要是：“后无追兵”的现状还没有得到根本改变，做大总量的压力不小；质量和效益低的现状还没有得到根本改变，产业转型升级的压力不小；城市建设滞后的现状还没有得到根本改变，扩容提质的压力不小。等等。对于这些工作薄弱环节，市政府将高度重视，采取有效措施切实加以解决。

2013年工作安排

今年是全面贯彻落实党的十八大精神的开局之年，也是我市经济社会继续保持平稳较快发展态势，逐步实现从量的积累到质的飞跃，推动“跨越赶超、科学崛起”的重要一年，做好今年工作意义重大。

当前，我市经济社会发展面临着很多有利因素。世界各国都出台相应政策，推动经济结构调整和发展模式转型，加快经济复苏。党的十八大和中央经济工作会议确定了我国经济继续稳中求进的总基调，出台了一系列稳增长、保民生的政策措施，为我国经济增长注入强大的发展动力，也为我市经济发展提供政策保障。省第十一次党代会提出要支持粤东西北地区地级市城区扩容提质、聚集发展、率先崛起等一系列战略部署，为我市经济社会发展，特别是做大做优做精城市提供了难得的发展机遇。同时，我市经过多年尤其是近几年的加快发展，交通体系、发展载体、产业基础等各方面条件已经得到了很大的改善和优化，发展条件日趋完善，后发优势日渐凸显。我们要坚定信心，抓住机遇，乘势而上，奋力开创经济社会发展新局面。

今年工作的总体要求是：坚持以邓小平理论、“三个代表”重要思想、科学发展观为指导，全面贯彻落实党的十八大、习近平总书记视察广东重要讲话、中央经济工作会议和省委十一届二次全会、市委五届四次全会精神，围绕“跨越赶超，科学崛起”的目标，以转变发展方式为主线，以生态文明建设为引领，改革创新，敢于担当，凝心聚力促发展，坚定不移惠民生，全力以赴保稳定，努力争创生态文明发展示范区，加快建设美丽幸福新云浮。

今年的主要预期目标是：地区生产总值增长15%；人均地区生产总值增长13.3%；地方公共财政预算收入增长20%；规模以上工业增加值增长29%；固定资产投资总额增长30%；社会消费品零售总额增长13%；居民消费价格涨幅控制在3.5%左右；城乡居民人均收入增长15.2%；单位GDP能耗下降3.89%。

为实现上述目标,今年的政府工作，重点要做好以下几个方面，具体做到“围绕一个核心，抓好两个关键，突出三个重点”：

一、紧紧围绕加快发展这个核心，努力促进经济提速增效

按照“加快发展、充分发展”的要求，坚持发展的路子不能偏、发展的目标不能变、发展的速度不能减、发展的重点不能松，努力盘活存量，扩大增量，提升质量，不断增强经济整体实力和内生源动力。

（一）牢牢抓住重点工程项目建设不放松。重点工程项目是加快云浮发展的希望所在，建设好、服务好这些项目是各项工作的重中之重。今年全市安排107项重点工程项目，计划总投资1366.74亿元，年度计划投资228.17亿元。全市上下要把它作为一切工作的生命线，全力组织攻坚，大力推进华润西江电厂、中材罗定水泥、华润罗定水泥、云浮青洲水泥、中国•云浮国际石材产业城、环科电解锰和硫酸项目、云浮风电等项目建设，做优做强石材、水泥、硫化工、不锈钢制品和电力五大百亿特色产业集群。要加快康利（云浮）石材工业产业园、珠海华策半导体LED产业基地、西江新城城市综合体、中顺洁柔罗定生产基地等项目建设。要全力推进云浮石材、罗定电子外贸、新兴不锈钢餐厨具三个转型升级专业示范基地建设。加强对本地企业特别是大型支柱企业的服务，扶持和鼓励企业增资扩产和转型升级，不断扩大优势主导产业规模。大力发展汽车零部件、电池电子、机械制造、纺织服装、高档生活用纸等产业。大力培育电池和电池机械制造产业集群发展，打造全国最大电池生产基地。支持房地产业健康发展。落实和完善引导民间投资政策，加大对中小微企业的扶持力度，鼓励中小微企业抱团发展，延长产业链条。

（二）牢牢抓住工业园区建设不放松。在完善园区基础设施和服务配套设施的基础上，推进园区扩容发展。努力建设一批发展潜力大的税源型、环保型项目，争取年内园区新动工、新建成工业项目投资额增长35%以上，其中佛山（云浮）产业转移工业园、佛山顺德（云浮新兴新成）产业转移工业园增长40%以上。不断提高园区贡献率，市重点工业园区对全市工业经济贡献率达到30%以上，实现产值增长25%，税收增长20%。大力扶持园区重点产业、主导产业加快发展，创建区域产业品牌，形成一批年产值超过150亿元的大型产业园区和年产值超过100亿元的产业集群。

（三）牢牢抓住招商引资工作不放松。充分发挥云浮的比较优势，结合云浮的产业基础、产业特点和产业规划布局，加快“双转移”创新提质，开展卓有成效的招商引资工作。认真研究国企、央企和龙头民企投向的新动态和新情况，加强与上级部门的沟通，争取更多的国资项目落户我市。发挥商会、协会、招商顾问及中介组织的作用，创新工作思路，加强沟通服务，瞄准全球500强企业和全国100强企业定向招商，结合我市主导产业开展产业链招商，争取年内引进全球500强企业1个、投资10亿元以上项目20个。办好第十届中国（云浮）国际石材科技展览会等经贸活动，提高相关产业的知名度，增强产业发展活力。

（四）牢牢抓住农业现代化发展不放松。依托广东温氏集团等农业龙头企业，扶持罗定“聚龙模式”发展，鼓励发展家庭农场，深入推进农业产业化发展到更多的镇、村，带动更多农户参与产业化经营。创新发展现代农业体制，提高农业经营规模和效益，新增省级农业龙头企业2家、市级农业龙头企业8家以上，新增农民专业合作社1000个以上。大力发展生态和经济林业产业，扎实推进油茶、南药、珍贵树种等特色产业发展，拓展林业经济增长点。围绕“一镇一业”和“一村一品”建设目标，大力发展生态农业和精品农业，发展新型农业生产经营模式，支持做大农产品流通和加工产业，提高农业发展的效益。创新农业发展思路，促进农业与二、三产业的整合和交融，推进农产品加工业和农业生态观光业的繁荣和发展。加强农业基础设施建设，扎实推进高标准基本农田建设，改善农业生产条件，促进农业增产、农民增收。

（五）牢牢抓住新兴产业培育发展不放松。加快培育电子商务、现代物流、信息服务、生物制药、保健养生等新兴产业，使幸福导向型产业成为云浮经济增长的新亮点。加快“智慧云浮”城市建设。加快数据容灾备份服务区、畜牧养殖物联网等项目建设；推进网络升级改造以及智能交通、智慧物流、食品安全、教育医疗等重点领域物联网应用示范工程建设。落实国家鼓励节能家电、小排量汽车等产品消费政策，推进“广货网上行”，支持高端新型电子信息、LED、新能源汽车、三网融合应用等高端产品市场应用和推广。积极搭建科技服务平台，加速科技成果转化，提升高新技术产值比重。全面提升地域文化的层次与品位，努力打造文化产业品牌，重点推进广东禅文化创意产业园、广东石艺文化创意产业园、南江文化创意产业园和信息创意产业园建设，办好第四届中国（云浮）石文化节、广东禅宗六祖文化节、第二届南江文化节、旅游文化节、罗定稻米文化节等活动，打响禅宗文化、石艺文化、南江文化和稻米文化品牌，促进文化与经济融合发展。完善旅游餐饮、休闲娱乐、保健养生等城市配套设施。整合全市旅游资源，完善各种旅游配套，打造生态旅游品牌，加快六祖故里生态旅游园区、蟠龙天湖景区、罗定龙湾生态旅游区、云安富林大云雾漂流度假区等旅游项目建设。

二、全力抓好交通基础设施建设和城市扩容提质两个关键

按照“科学规划、有序推进”的原则，全面改善云浮的区位条件、投资环境和生活环境，全面优化城市发展空间和功能布局。

（一）全面加快交通基础设施建设，突破制约发展瓶颈。加快交通基础设施建设是云浮融入珠三角、借力珠三角实现加快发展的关键所在。继续以“七三三三一”工程为重点，以完善交通网络建设为抓手，全力加快交通基础设施建设，全年计划投入60.77亿元。确保云岑高速公路全线建成通车，加快阳罗高速、江罗高速、南广高铁建设，争取汕湛高速年内动工建设，争取广云高速思劳立交、高村立交年内建成。争取年内完成三茂铁路云浮支线至云浮新港铁路、西江都杨星晖码头、造船厂和物流园等项目的前期工作。加快云杨公路（新城快线一、二期）、沿江路二期、新城至云安组团连接线（站前路）、新城至腰古组团连接线、罗定市环市路、新兴县二环路西段、省道369线路面改造、省道279线路面改造等公路建设。加快汕湛高速簕竹至湛江段的前期工作，确保“十二五”末动工建设。做好怀罗高速、罗信高速、肇云轻轨等项目储备，争取早日落地建设。加快南江口码头建设，积极利用国家放开低空航线的契机，加快重新启动罗定机场。通过完善公路、铁路、水路、物流、空运和综合运输站场枢纽网络，逐步实现多种交通方式的无缝对接，构建综合交通运输体系，不断改善交通区位条件，为经济社会发展注入强劲动力。

（二）全面加快城市扩容提质，破解城市发展短板。以新型城市化发展理念，处理好城市扩容与提质的关系。在空间上把城市的架构拉开，扩大城市容量; 在内涵上把城市的品质做优，提升城市品位。通过扩容提质，促进全市在环境质量、聚集能力、承载功能、居住条件、风貌特色、管理服务等方面全面提升。

加快云浮新区规划建设。打破行政区域界限，推进城区扩容提质，提升中心城区的辐射带动力。重点规划建设881.81平方公里的云浮新区，重中之重是科学运用好西江新城规划国际竞赛成果，加快80平方公里核心区的规划建设，人口发展到40万人。将西江新城建设成为全市政治、经济、文化、教育中心，建设成为具有岭南山水、广府文化特色的宜居、宜业、宜游、宜休闲的幸福新城。通过云浮新区的崛起，引领带动全市域的新型城镇化建设。年内主要是加快西江新城大环路前期工作，争取年内动工建设市民服务中心门前大道；加快西江新城低碳环保新型公交项目建设，年内完成新城至云城城区增程式公交电车项目前期工作；加快推进市民服务中心及新区服务中心、中央商务区，以及华立城市学院、意大利风情小镇、碧桂园等重大项目的规划建设。加快西江新城水利项目的规划建设和防洪治涝工程建设，夯实城市发展基础。

提升中心城区品位。强力推进市中心城区市政设施的改造和完善。以整治、拓宽道路和打通断头路为重点，努力贯通罗斗岗路、金山路南段、绿屏路南段、河南东路振华小学段、浩林西路、金丰路，加快推进富民路、牧羊路等市政道路改造。推进市中心城区周边石材企业迁移整治，年内完成牧羊路路段石材企业搬迁。加快市中心城区“三旧”改造和蟠龙天湖片区改造。加强对城市交通、供水、供电、供气、通讯、环卫等领域以及中心城区、城乡结合部的管理，增强城市综合载体功能。尽快理顺城市管理体制机制，加强城市综合执法，实现城市管理重心下移和城市管理新突破。同时，支持罗定市泷州新城建设，打造集商贸居住、休闲娱乐于一体的多功能新区。其它县区要抓好一批扩容提质项目建设，努力提高城市可持续发展能力。

推进城镇化建设。按照城镇化建设的要求，继续完善主体功能区规划，构建科学合理的城市化格局、工业发展格局、农业发展格局、生态安全格局，保护好云浮的青山绿水，促进生产空间集约高效、生活空间宜居适度、生态空间山青水秀。加快专业镇建设，根据当地主导产业和特色经济发展及城镇发展的需要，合理确定产业方向、城镇规模和设施配套，加紧做好专业镇主导产业发展规划和城市规划建设的调整和修编。合理控制环境容量，提高科学建设标准，加强资源保护利用和空间管制，避免重复建设和无序发展等问题。以中心镇建设为突破口，扎实推进和谐秀美乡村、宜居社区和名镇名村建设，既在农村基础设施建设、卫生环境改善上下功夫，又要注重在基本公共服务和社会保障等方面统筹推进。

三、突出抓好综合改革、民生福祉和干部作风建设三个重点

通过深化改革和转变作风，释放发展的巨大潜力、源头活力和做好民生工作的持久动力，使人民群众得到红利、收到实惠。

（一）进一步深化综合改革。紧扣“三农”发展，深化产业带动、土地流转、金融支撑、协同共治、生态文明村“五位一体”的农村综合改革，建设全省、全国农村改革试验区。创新金融服务，加快金融改革创新步伐，全面落实农村金融改革创新综合试验区各项工作，完善地方金融体系。大力支持金融机构建设，推动农村信用社改制为农村商业银行，加大国内外金融机构引进力度，年内争取落实2家以上金融机构进驻我市，探索试行“政银保”合作模式涉农贷款，加快温氏集团整体上市步伐。积极发展涉农保险，逐步建立广覆盖的涉农保险保障体系，增强农业抵御自然灾害能力。大力发扬“诚信、包容、实干”的云浮精神。推进社会管理创新，扎实抓好信用云浮建设、培育和发展自然村乡贤理事会、弘扬优秀传统文化促进社会管理创新等省社会创新项目，确保取得新成效，推动和谐社会建设。深化社会组织体制改革工作，着力培育和发展社会组织，促进社会协同共治。

（二）进一步增进民生福祉。各位代表，我代表市人民政府承诺，今年继续集中力量办好创业就业、教育事业、医疗卫生、文化体育、农村发展、助困扶残、宜居城乡、社会保障、生态工程、品牌云浮和稳价惠民等“十件民生实事”，让发展的成果惠及广大群众。

抓好教育创强工作。统筹推进幼儿教育、义务教育、特殊教育、普通高中教育、职业教育、远程开放教育等各类教育事业，加快推进市教育园区建设，合理调整学校布局和加快规范化学校建设，缩小城乡教育差距，促进教育均衡发展。加强教师队伍建设，引进一批高层次教育人才，强化教学管理，提升教育质量，力争高考本科上线率有新突破。提升罗定职业技术学院办学水平。加快推进云浮实验学校建设。邓发中学要扩规模，提水平，打造成为全市一流的重点中学。

抓好就业和社会保障。大力支持创业促进就业，千方百计增加就业岗位，提升就业质量。实施技能提升培训工程，积极推动农村劳动力“三化”转移就业，壮大高技能人才队伍。统筹城乡就业，不断完善就业公共服务体系，年内实现镇（街）人力资源社会保障所全覆盖，确保城镇登记失业率控制在全国平均水平以内。全面推进社会保险扩面征缴，确保年内城乡居民基本医疗保险覆盖率达99%以上，城乡居民社会养老保险参保率达75%以上。不断完善城乡居民医疗保险引入市场机制办法，积极实施社会保障“一卡通”工程，探索市外就医异地结算工作，提升社会保险经办服务和信息化水平。加大保障性住房建设力度，改造建设农村低收入住房困难户住房2815户。加强云浮福利院、罗定市社会福利服务中心等社会福利事业建设，完善社会救助体系。推进新一轮扶贫开发工作，认真做好城乡低保、救灾救济等工作，落实双拥优抚安置政策，努力推动社会保障由补缺型向普惠制转变。

抓好文化体育卫生等公共服务建设。牢固树立“文化民生”理念，不断完善城乡公共文化服务网络，争取年内完成全市镇（街）综合文化站建设，实现行政村级文化室建设工程全覆盖。加快完善体育配套服务设施，推进全民健身运动。全面深化医药卫生体制改革。抓好市、县、镇、村四级医疗卫生机构建设，健全城乡医疗卫生服务体系。深入实施基层医疗卫生机构基本药物和一般诊疗费制度，切实减轻城乡居民医药费用负担。推进创建国家卫生城市和省级卫生镇、卫生村活动，抓好中医药强市和南药基地建设，加快推进市人民医院二期建设、云浮中医院整体搬迁和市妇幼保健院新院建设等工作。加强医疗卫生队伍建设，全面提升医疗卫生服务质量和水平。推动3万户农村卫生改厕。

抓好生态文明建设。以生态景观林、森林碳汇和森林进城围城三大重点生态工程建设为重点，全面打造生态优美幸福云浮。巩固和发展省园林城市成果，提升新老城区绿化美化水平。消灭11.2万亩宜林荒山，改造44.1万亩疏残林、纯松林和布局不合理的速生林，全面构建稳定的生态安全屏障。加强环境保护，提高准入门槛，限制高能耗和污染项目进入。加强水泥、硫化工等高能耗、高污染产业的监管，确保其达标排放。大力发展循环经济，巩固循环经济试点成果。节约集约利用资源，支持节能低碳产业和新能源、可再生能源发展，促进生产、流通、消费过程的减量化、再利用和资源化。扎实推进生态文明村建设，实行领导干部直接负责1条自然村的工作制度。继续开展“大清洁，乡村美”农村清洁专项活动，加快农村垃圾处理设备设施建设，实现“一镇一站、一村一点”建设达标，确保全市农村垃圾收集清运处理率达60%以上。大力实施中小河流治理、村村通自来水、治洪治涝保安等民生水利建设。创新人口计生工作机制，全面开展免费孕前优生健康检查，提高人口素质。稳定低生育水平，完成人口与计划生育目标管理责任制工作任务。

抓好“平安云浮”创建活动。加强城乡社区自治和服务组织建设，做好流动人口和特殊人群服务管理。健全城乡一体化现代治安防控体系，严密防范和依法打击各种违法犯罪活动，提高人民群众的安全感。开展食品药品监管和安全生产隐患专项治理，坚决遏制重特大事故发生。启动全国农产品质量安全监管示范市创建工作，在全市各镇（街）全面建立农产品质量安全监督服务机构。健全突发公共事件预警和应急处置机制，提高防灾减灾和应对突发事件的能力。建立覆盖城乡的法律服务体系，加强法律援助工作。支持工会、共青团、妇联、红十字会、慈善会等人民团体和组织发挥稳定桥梁作用。提高双拥水平，加强人民防空、民兵预备役和部队建设。

（三）以作风转变推动工作落实。坚决落实党中央“八项规定”，以作风的转变推动工作落实、带动效能提升、促进服务型政府建设。强化依法行政，积极推进“六五”普法和法治政府建设进程，加强执法公开、政务公开、行政复议和行政应诉工作，提高依法行政水平。认真落实市委、市人大及其常委会各项决议决定，主动向人大及其常委会报告、向政协通报工作，自觉接受人大的依法监督和政协的民主监督，切实提高建议、提案的办理质量。加快推进以行政审批为重点的体制机制改革，健全便民服务体系，深化代办服务，创新代办模式，完善网上审批。加强对工程建设、土地出让、资金管理等重点领域的投资服务，加大对公共资源交易的监管力度。倡导艰苦奋斗、勤俭节约之风，从严控制行政经费和一般性支出，因公出国（境）经费、公务用车购置及运行费用、会议费用、公务接待费支出保持零增长。坚持从严治政，建立健全惩治和预防腐败体系，认真落实党风廉政建设责任制，严格执行廉洁从政各项规定，严肃查处违纪违法案件，切实纠正各类损害群众利益的不正之风，树立公正廉明的政府形象。

各位代表，新的征程充满新机遇，面临新挑战，孕育新希望。让我们在市委的正确领导下，深入贯彻落实科学发展观，紧紧依靠全市人民，以更加开阔的眼界、更加务实的作风、更加昂扬的斗志，开拓创新，埋头苦干，为实现“跨越赶超、科学崛起和美丽幸福新云浮”而努力奋斗！
